# Development of a Web Application for Simulating Plasma Drug Concentrations in Patients with Zolpidem Intoxication

**DOI:** 10.3390/pharmaceutics16050689

**Published:** 2024-05-20

**Authors:** Hwa Jun Cha, Sungpil Han, Kwan Cheol Pak, Hyungsub Kim

**Affiliations:** 1 Department of Pharmacology, College of Medicine, The Catholic University of Korea, Seoul 06591, Republic of Korea; clex9604@catholic.ac.kr (H.J.C.); shan@catholic.ac.kr (S.H.); 2 PIPET (Pharmacometrics Institute for Practical Education and Training), College of Medicine, The Catholic University of Korea, Seoul 06591, Republic of Korea; 3 Yuhan Corporation, Seoul 06927, Republic of Korea; kcpak4175@yuhan.co.kr; 4 Department of Paramedicine, College of Health Sciences, Eulji University, Seongnam 13135, Republic of Korea

**Keywords:** zolpidem, drug intoxication, pharmacokinetics, simulator, web application

## Abstract

Zolpidem is a widely prescribed hypnotic Z-drug used to treat short-term insomnia. However, a growing number of individuals intentionally overdose on these drugs. This study aimed to develop a predictive tool for physicians to assess patients with zolpidem overdose. A population pharmacokinetic (PK) model was established using digitized data obtained from twenty-three healthy volunteers after a single oral administration of zolpidem. Based on the final PK model, a web application was developed using open-source R packages such as rxode2, nonmem2rx, and shiny. The final model was a one-compartment model with first-order absorption and elimination with PK parameters, including clearance (CL, 16.9 L/h), absorption rate constant (K_a_, 5.41 h^−1^), volume of distribution (V_d_, 61.7 L), and lag time (ALAG, 0.394 h). Using the established population PK model in the current study, we developed a web application that enables users to simulate plasma zolpidem concentrations and visualize their profiles. This user-friendly web application may provide essential clinical information to physicians, ultimately helping in the management of patients with zolpidem intoxication.

## 1. Introduction

Insomnia is conceptualized to be hyperarousal at night caused by the inability to switch off arousal-related circuits, which may be a risk factor for various psychiatric disorders such as depression, anxiety, and substance use disorders [1]. The estimated prevalence of insomnia is over 10% of the adult population, and patients with insomnia complain of difficulty with sleep onset, sleep maintenance, or early awakenings. Several factors have been related to insomnia, including abnormal glucose metabolism, increased activation of the autonomic nervous system, decreased GABA (gamma-aminobutyric acid) levels, and reduced melatonin secretion [2,3]. To treat insomnia pharmacologically, several positive allosteric modulators of GABA_A_ receptors, such as benzodiazepines (triazolam, estazolam, temazepam, quazepam, and flurazepam) and Z-drugs (zaleplon, zolpidem, and zopiclone) were approved and widely used [1,4]. However, because of withdrawal effects, tolerance, and rebound insomnia from benzodiazepines, Z-drugs are prescribed as a first-line hypnotic agent. Among the Z-drugs, approximately 3.8 million adults are prescribed zolpidem annually in the United States, and zolpidem has been ranked first in the global market share for more than 10 years [5,6,7]. Due to this widespread use, a variety of formulations are available, including an oral spray and sublingual tablets. Different from the benzodiazepines, zolpidem binds selectively to alpha subunits of benzodiazepine-sensitive GABA_A_ receptors [1]. Pharmacokinetically, zolpidem (*N*, *N*-6-trimethyl-2-[4-methylphenyl] imidazo [1,2-α] pyridine-3-acetamide hemitartrate) has a relatively short half-life of 1.5–2.5 h after a single oral dosing and it is predominantly metabolized to inactive metabolites by CYP3A4 isoform in the liver [8,9]. The bioavailability is 70% with plasma peak within 2 h. Zolpidem is associated with several adverse effects affecting more than 1% of individuals, such as central and peripheral system depressant effects (drowsiness, dizziness, headache, ataxia, and euphoria), nausea, vomiting, myalgia, or diplopia. In rare instances, more severe complications such as hallucination, abnormal thinking, or respiratory depression can occur. The recommended initial dose is 5 mg or 10 mg of immediate-release formulation for men before bedtime. Women, the elderly, and patients with decreased liver function receive the lower dose of 5 mg [10,11]. In post-marketing reports of overdose with zolpidem alone or in combination with CNS-depressant agents, increased risks of consciousness ranging from drowsiness to coma and fatal results have been reported [10,12]. In terms of clinical toxicology, the therapeutic range of plasma zolpidem concentration is 80–200 ng/mL, and the toxic, comatose, and fatal thresholds are over 500, 1500, and 4000 ng/mL, respectively [13]. In cases of zolpidem overdose, treatment typically involves gastric lavage, and activated charcoal administration may be considered in the early stages of overdose to minimize further absorption of zolpidem, as well as supportive care. In severe cases, flumazenil, a benzodiazepine receptor antagonist, may be used to reverse the sedative effects of zolpidem.

Suicide is a serious social and health problem, and South Korea’s suicide rate is the highest among the OECD countries [14]. Among suicide methods in South Korea, acute poisoning is the most common, and the primary causative substances frequently involved in drug intoxication are benzodiazepines, zolpidem, and antipsychotics [15,16]. From 2014 to 2016, the National Forensic Service found that drugs were the most common causative substances among 699 cases of suspected poisoning in Seoul and its metropolitan area, 142 of which involved a combination of two or more drugs, and zolpidem was detected most frequently in the Republic of Korea [17]. Acute drug intoxication, especially sedation- and sleep-inducing drugs, is the most common reason for emergency room (ER) visits due to drug overdose-related suicide attempts. Patients who visit the ER due to drug intoxication often lose consciousness and experience life-threatening complications, such as respiratory depression. Therefore, it is difficult to assess and treat patients, because it is difficult to confirm whether the drug has caused poisoning, even if poisoning is suspected. Moreover, the blood concentration of a specific drug cannot be measured quickly in any medical institution.

Pharmacometrics is a field that contributes to drug development by minimizing errors in initial dose determination and safety prediction through population pharmacokinetic (PK) modeling by non-linear mixed-effect modeling (NONMEM) using early clinical trial data [18]. NONMEM is software that explains observed data through the model by addressing population means and interindividual variations [19]. Through the mean and interindividual variation of the parameters estimated in the model, each individual’s observed data are described relative to the population means, accounting for differences between individuals. By expanding these scientific advances in emergency medicine, we aimed to develop a new tool to estimate the blood concentration of zolpidem, a frequently prescribed sleep drug, in patients with acute zolpidem intoxication, ultimately helping to quickly assess and treat patients in the ER. In the present study, we attempted to establish a population PK model using digitized data from South Korean volunteers and developed a web application to predict the plasma concentration of zolpidem in patients with acute intoxication.

## 2. Materials and Methods

### 2.1. Study Design and Data

An open-label, one-period, parallel-group, single-dose study was conducted to investigate the pharmacokinetic profile of 10 mg zolpidem (film-coated tablet, immediate-release formulation) in 30 healthy Korean volunteers (15 subjects for each sex) registered with the Clinical Research Information Service (KCT0003934; date of registration: 28 August 2018) [20]. Blood samples for PK analysis were collected using K_2_–EDTA tubes at the following time points: pre-dose (0 h) and post-dose at 0.25, 0.5, 0.75, 1.0, 1.5, 2, 3, 4, 6, 8, and 12 h. Using WebPlot Digitizer, we extracted concentration data from a graph of individual plasma profiles for population PK modeling [21,22]. After data extraction, a non-compartment analysis (NCA) was conducted to compare the digitized dataset against the original PK data using the R NonCompart package (version 0.7.0) [23,24]. NCA parameters such as C_max_, area under the curve (AUC), clearance (CL/F), slope of the elimination phase (lambda z), terminal half-life, volume of distribution (V_d_/F), and T_max_ were compared, and AUC was calculated using the linear-up and log-down method.

### 2.2. Population PK Model

Population PK analysis was performed using non-linear mixed-effects modeling (NONMEM software, version 7.5.0 with a subroutine ADVAN14; ICON Development Solutions, Ellicott City, MD, USA) [25]. One-, two-, and transit-compartment models with first- or zero-order absorption kinetics, including absorption lag time, were tested. Population PK parameters, interindividual variability, and residual variability were estimated using the first-order conditional estimation with interaction (FOCE-I) method. The interindividual variability of each parameter was described using a lognormal distribution.

P_i_ = θ_pop_ · exp(η_i_)

P_i_ is the individual parameter, θ_pop_ is the typical parameter value for the population, and η_i_ represents the interindividual variability, which follows a normal distribution with a mean of zero and variance of ω^2^. The residual variability was modeled as a proportional error.

DV_ij_ = DV_ipred.ij_ · (1+ ε_prop.ij_)

DV_ij_ is the jth measured concentration in ith individual, DV_ipred.ij_ is the jth model prediction in ith individual, and ε_prop.ij_ represents the proportional residual variability which follows a normal distribution with a mean of zero and variance of σ^2^. Covariate analysis was performed, particularly in terms of sex, based on a previous report [26].

Graphical assessments and statistical analyses of each model were performed to evaluate the significance of the model improvements. Differences in the objective function value between the two fits of the hierarchical models were approximately chi-squared distributed with degrees of freedom equal to the difference in the number of parameters between the models. A significance level of 0.05 was considered for the likelihood ratio test during model building, meaning that a drop of >3.84 in the objective function value after adding a single model parameter was deemed a statistically significant improvement in the model. Additionally, basic goodness-of-fit plots, including observed data versus population prediction, observed data versus individual prediction, conditional weighted residuals (CWRES) versus population prediction, and CWRES versus time after the dose, were assessed.

### 2.3. Model Evaluation

The visual predictive check (VPC) and bootstrap methods were used to evaluate the final PK model. One thousand bootstrap datasets were generated by resampling the participants from the original dataset, and the median and 95% confidence intervals of the parameters were calculated to compare the parameter estimates of the final model. Using the 1000 simulated datasets obtained from the Monte Carlo simulation. R software version 4.3.3 (R Foundation for Statistical Computing, Vienna, Austria) was used for data preparation, graphical diagnosis, and statistical analyses [27].

### 2.4. RxODE Conversion and Simulation

After the final population PK modeling, two open-source R packages were utilized for conducting simulations in the R environment. These included nonmem2rx (version 0.1.3) [28], and rxode2 (version 2.1.0) [29,30]. The role of the nonmem2rx package is to convert the NONMEM control stream into RxODE syntax. In this process, the package reads NONMEM files such as the output file and dataset and conducts the comparative analysis to show how well the conversion was conducted by comparing the population prediction (PRED), individual prediction (IPRED), and individual weighted residuals (IWRES) of NONMEM and converted RxODE model. The rxode2 package was used to perform and manage the simulation within R. It facilitates fast and efficient simulations of ordinary differential equation-based models in various user-defined dosing regimens.

### 2.5. Development of A Web-Application

The web application was developed using an open-source shiny R package that provides the framework of user-interactive web applications [31]. Users can define various dosing regimens for zolpidem, and the application then simulates and predicts the drug’s plasma concentration profile and pharmacokinetic parameters. The user interface is designed to be user-friendly, allowing for easy adjustments of input values and facilitating the straightforward visualization of results, making it clear to see the impact of different dosing regimens on zolpidem’s PK [32].

## 3. Results

### 3.1. Pharmacokinetic Analysis

**Figure 1:**
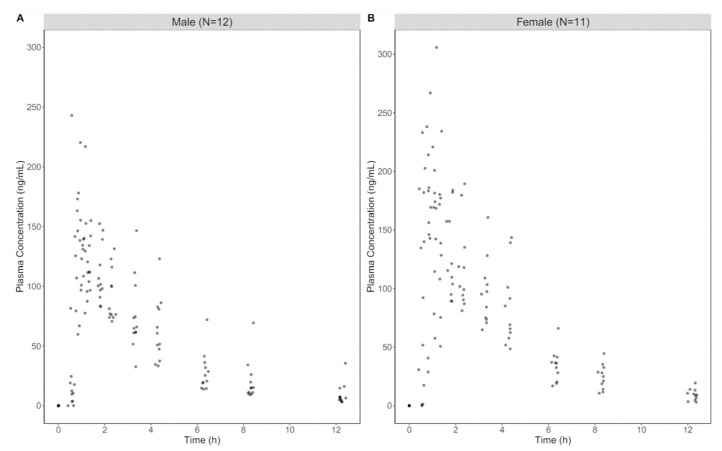
Observed data of plasma zolpidem concentrations. The raw data were digitized using the WebPlot Digitizer from the research report available on the Ministry of Food and Drug Safety website. (**A**) Male participants (N = 12); (**B**) female participants (N = 11).

Data from seven subjects were excluded from the Ministry of Food and Drug Safety (MFDS) report due to errors such as duplicated graphs [33]. Upon closer examination, it was found that the pharmacokinetic profiles of these subjects were identical to other participants’ graphs. Consequently, data from 23 subjects (12 males and 11 females) were used for the population pharmacokinetic analysis in this study. Demographic characteristics of 23 subjects could not be summarized due to unavailability of the individual data. However, demographic results are expected to be similar to the original study with a smaller standard deviation. Mean age, height, and weight of male participants (N = 15) were 30.2 ± 5.9 years, 173.5 ± 5.5 cm, and 73.1 ± 7.6 kg, and 29.9 ± 6.2 years, 159.9 ± 5.6 cm, and 54.7 ± 3.9 kg for female participants (N = 15), respectively [33]. NCA was performed to compare the similarity of the data. The results were similar, with no statistically significant differences in any PK parameters between the digitized and original data (Table 1). Digitized concentration data for zolpidem are shown in Figure 1.

### 3.2. Population PK Model

A one-compartment linear model with delayed onset best described the plasma concentrations after the oral administration of zolpidem. The introduction of a parameter reflecting time delay (ALAG) in the PK model significantly improved the model (ΔOFV = 25.3). No other structural models, including various absorption models that explain the early absorption phase, showed any significant improvement. However, the result of the covariate analysis showed that sex was not significant in the current study. The PK parameter estimates for the final model are summarized in Table 2. The population parameter estimates of the final model were close to the median values of the parameter estimates from 1000 bootstrap replicates, and their 95% confidence intervals demonstrated the robustness of the final model (Table 2).

**Figure 2:**
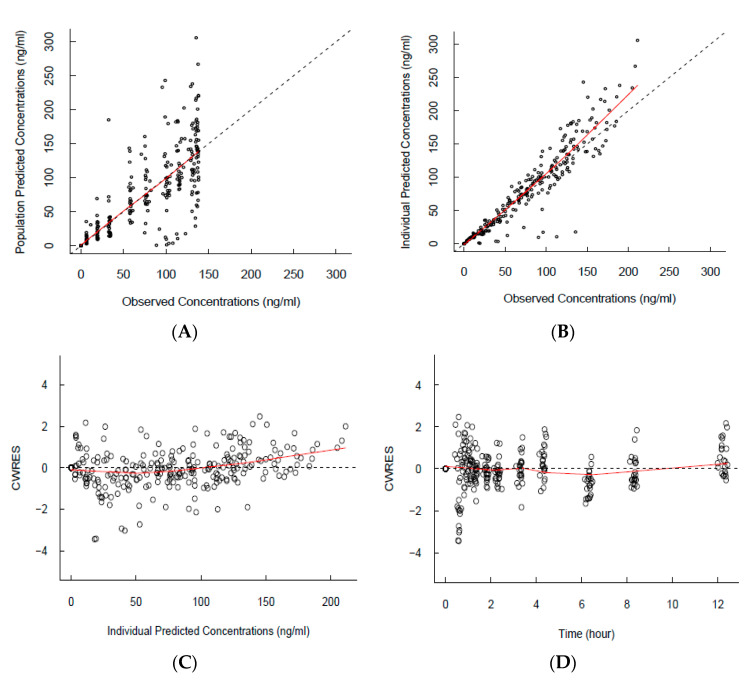
Basic goodness-of-fit plots of the final population pharmacokinetic model. Circles indicate the observed/predicted blood concentrations. The black dashed lines indicate the identity or zero line. The red lines indicate the locally weighted scatterplot smoothing line. (**A**) Scatterplot of PRED (population predictions) versus observed concentrations. (**B**) Scatterplot of IPRED (individual predictions) versus observed concentrations. (**C**) Scatterplot of conditional weighted residuals (CWRES) vs. individual predictions. (**D**) Scatterplot of CWRES vs. time after dosing.

**Figure 3:**
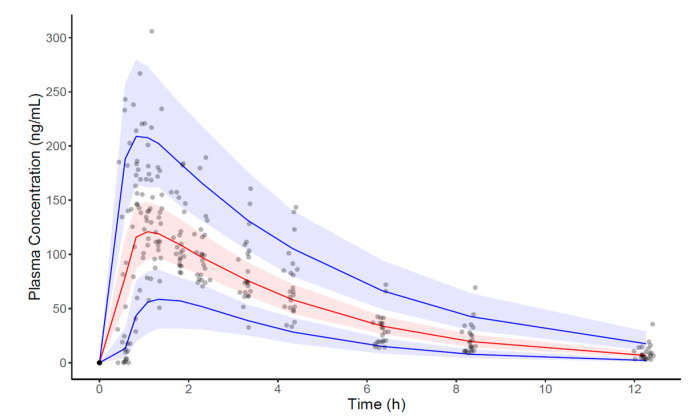
Visual predictive check (VPC) for zolpidem concentrations using the final PK model. Observed concentrations are depicted as gray dots. The figure presents the VPC with the prediction intervals for the 5th, 50th, and 95th percentiles of simulations from bottom to top. The solid lines correspond to 5th, 50th, and 95th percentiles. The blue shaded areas correspond to 95th confidence interval of the 5th and 95th percentile. The red shaded area corresponds to 95th confidence interval of the 50th percentile.

The basic goodness-of-fit plot demonstrated adequate predictive performance, and no specific trend was observed, as shown in Figure 2. In addition, the visual predictive check plot showed adequate predictive properties of the final model, even though slight underprediction was observed (Figure 3).

### 3.3. RxODE Conversion and Simulation

After developing the population PK model, it was converted into RxODE syntax using the nonmem2rx package. This conversion allowed us to easily translate the PK model into R, laying the groundwork for developing the base model. The sampling time for the simulation was specified by the PK characteristics of zolpidem. As shown in Table 1, while the median T_max_ values were between 0.75 and 2.6 h, the T_max_ for females extended up to 3.36 h. To accommodate this observed variability and optimize more accurate simulation, we specified the sampling time to be more frequent up to 4 h to monitor the extended absorption phase. Beyond this, the sampling intervals were widened to reduce the overall simulation time. This simulation model with adjusted sampling times was used as the base model for the shiny web application.

### 3.4. Zolpidemsim: Web Application

**Figure 4:**
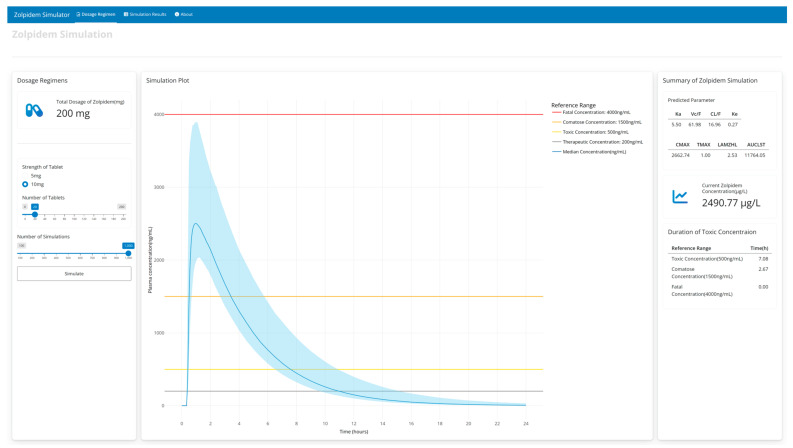
An example of the web application simulated in the case of a 200 mg zolpidem overdose (20 tablets of 10 mg zolpidem).

Using the shiny package in R, a web-based user-interactive pharmacokinetic simulation application (Zolpidemsim) was constructed. After developing the population PK model, it was converted into RxODE syntax using the nonmem2rx package. This conversion allowed us to easily translate the PK model into R, laying the groundwork for the development of web applications. The utilization of rxode2 allows for swift and efficient simulations across various dosing scenarios, providing a rapid and reliable computational platform crucial for dynamic pharmacokinetic modeling and analysis. This application consists of three panels, “Dosage regimen”, “Simulation results”, and “About”, and is available online for a responsive simulation (https://pipettox.shinyapps.io/Zolpidem (accessed on 13 April 2024)). In the “Dosage regimen” panel, users can define their preferred dosage regimens via various sidebars, such as dose strength, number of tablets, and number of simulations. Based on the user input, the total dose of zolpidem is automatically calculated and displayed on top of the sidebar. The simulation is initiated by clicking the “Simulate” button. Then, users can view the simulation plot which visually represents the outcomes including 5th, 50th, and 95th percentiles along with the reference range of zolpidem concentration. Also, a summary of the simulation such as predicted parameters, current zolpidem concentration, and duration of toxic concentration is displayed. For a comprehensive analysis, users can navigate to the “Simulation results” panel, which shows detailed simulation outcomes This includes both population PK parameters and NCA results, such as C_max_, T_max_, AUC, and half-life. In addition, this application illustrates the duration for which plasma concentrations remain above therapeutic, toxic, and fatal thresholds. A representative example of the simulation in a patient administered 200 mg of zolpidem is displayed in Figure 4.

### 3.5. Simulation Results at Various Doses

**Figure 5:**
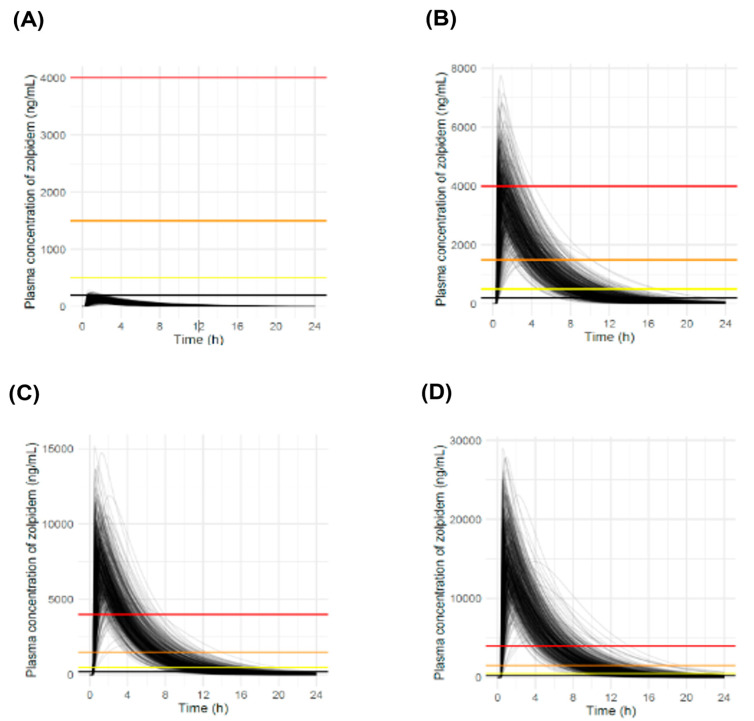
Simulated PK profiles of zolpidem after oral single dosing (N = 500): (**A**) 10 mg zolpidem; (**B**) 280 mg zolpidem; (**C**) 560 mg zolpidem; (**D**) 1120 mg zolpidem. The colored lines indicate concentration thresholds: the red line represents the fatal concentration threshold (4000 ng/mL), the orange line represents the comatose concentration threshold (1500 ng/mL), and the yellow line represents the toxic concentration threshold (500 ng/mL).

Demonstrative simulations using the application were conducted at four different doses to evaluate the PK profiles of zolpidem. These regimens included a single dose of 10 mg, the recommended daily dose of zolpidem as a reference [10,12]. An additional simulation was performed at a dose of 280 mg, equivalent to the total amount prescribed for four weeks, contained in one package. We then simulated doses of 560 and 1120 mg, which were equivalent to two and four packages, respectively (Figure 5). These doses were selected based on the total dose in a single package and the regulatory context in South Korea, where zolpidem is recommended for up to 28 days.

As shown in Figure 5, the simulated profiles of a single administration of 10 mg of zolpidem remained mostly below the therapeutic threshold and did not reach the toxicity threshold. However, at higher doses, the plasma zolpidem concentration remained above the toxic and comatose levels for a considerable period. In the case of 280 mg dosing, fatal concentrations were simulated in 21.4% of the total simulations within 1 h after dosing. As expected, a remarkable increase was noted at a dose of 1120 mg, where the fatal concentration level was maintained for over 3 h in 98.4% of the total simulations (Table 3).

## 4. Discussion

Previous clinical trials using zolpidem demonstrated that its plasma concentration increased rapidly and decreased in the PK profile [22,34]. According to a PK/PD study, this was adequately explained by the sigmoid E_max_ model based on ligand-binding theory [35]. Although zolpidem is known to be a relatively safe compound owing to its short duration of action, it may cause life-threatening complications such as anaphylaxis or respiratory depression upon overdose. In fact, coadministration of zolpidem with alcohol, H1 antihistamines (first generation), benzodiazepines, phenobarbital, and opioids increased the risk of CNS depression and respiratory depression due to the drug–drug interaction (DDI) [10].

To develop a web application for simulating plasma zolpidem concentrations in patients with drug intoxication, we constructed a population PK model using digitized data from 23 healthy volunteers (12 males and 11 females). This website (https://pipettox.shinyapps.io/Zolpidem (accessed on 13 April 2024)) is mainly targeted at physicians who treat patients with drug intoxication in the ER. Therefore, we focused on the maximum concentration (C_max_) and time to reach the therapeutic level, which is related to severity and recovery time, respectively. Additionally, our simulation results at various doses showed a direct correlation between the zolpidem dose and the duration for which the plasma concentrations remained above critical levels. This finding suggests that as the dose increases, the duration of critical plasma concentrations increases, indicating an increased potential for adverse effects.

In our study, we chose to run the simulations in the R environment because of its robust suite of tools for analyzing and visualizing data, enhanced by the release of the shiny package, which allows easy development of interactive web-based interfaces. These capabilities make R an ideal platform for pharmacokinetic simulations that benefit from real-time interaction and data representation. Building upon R’s capabilities, we incorporated the rxode2 and nonmem2rx packages, enhancing the simulation process for ODE-based PK/PD models. This collaboration between these two packages offers distinct advantages over other available packages such as mrgsolve [36], PKPDsim [37], and deSolve [38]. The nonmem2rx package enables the direct conversion of NONMEM models to RxODE syntax, which markedly simplifies the simulation process by reducing manual scripting. This reduces the risk of human error and significantly saves the time required to set up the simulation. Additionally, the rxode2 package enables specification of dosing scenarios and offers more rapid execution times for these simulations than other packages [31]. Thus, integrating rxode2 and nonmem2rx into our R environments not only enhances our capacity for sophisticated PK/PD simulation but also ensures a more efficient, accurate, and user-friendly experience, ultimately contributing to the robustness and reliability of pharmacokinetic analyses.

Drover et al. reported that a one-compartment model with first-order absorption and elimination phases described PK kinetics and none of the covariates produced a significant improvement [34]. In contrast, Saldanha et al. reported that a two-compartment model with lag time, first-order absorption, and linear elimination best described zolpidem cases [22]. Thus, zolpidem is suitable for various PK models, depending on the concentration data of individuals. In the original study, which included 30 subjects, a two-compartment model combining the Erlang-type and zero-order absorption models was constructed as the basic PK model [33]. However, in this study, the plasma concentration of zolpidem was best described by a one-compartment model without a covariate (N = 23). Although exposure to zolpidem was approximately 30% higher in females than in males, according to a previous report, our results demonstrated that sex was not selected as a covariate. None of the PK parameters from the NCA was significantly different between males and females, except for the volume of distribution (*p* < 0.05). Although this study used digitized data from a limited number of participants, our results are meaningful and reasonable for evaluating patients with intoxication from an emergency medicine perspective.

This study had several limitations. In cases of drug intoxication, patients typically take dozens to hundreds of tablets, which can interfere with absorption. As a result, the maximum concentration (C_max_) of the drug in the bloodstream would decrease owing to the low dissolution or absorption delay, as mentioned previously. In addition, patients with acute zolpidem overdose occasionally take it with alcohol or other psychotropic drugs, which can lead to a more severe condition than expected from the plasma drug concentrations due to the drug interaction [9]. Because of the short duration of an immediate-release formulation, a controlled-release (CR) formulation has been prescribed to improve sleep maintenance. Therefore, further studies applicable to various clinical situations, including DDI and CR formulation, are needed. Another limitation was the inability to assess the influence of demographic factors on the pharmacokinetics of zolpidem due to the unavailability of individual demographic data other than sex. However, based on the methodology established in the present study, although it uses digitized data, this approach is expected to expand in the future as a platform capable of easily simulating the blood concentrations of acutely intoxicated drugs.

## 5. Conclusions

We developed a web application that enables physicians to perform various easy-to-use simulations of zolpidem dosing using a web browser. This web application might help understand how plasma zolpidem concentration changes over time and how long toxic zolpidem concentration levels persist. Thus, this web tool may be helpful in the clinical assessment and management of patients with zolpidem intoxication in the ER.

## Figures and Tables

**Table 1:** Non-compartment analysis results of digitized data.

Parameters	Male		Female	
	Original (N = 15)	Digitized (N = 12)	Original (N = 15)	Digitized (N = 11)
C_max_ (ng/mL)	161.21 (46.74)	162.72 (47.04)	187.64 (59.44)	189.22 (59.85)
AUC_0–12h_ (ng h/mL)	537.9 (202.3)	542.5 (197.5)	696.9 (221.7)	688.1 (221.3)
CL/F (L/h)	19.76 (7.25)	18.88 (6.06)	14.72 (4.07)	14.93 (4.22)
Lambda z (1/h)	0.27 (0.09)	0.22 (0.06)	0.23 (0.03)	0.24 (0.04)
Half-life (h)	2.93 (1.14)	3.58 (1.10)	3.05 (0.35)	3.02 (0.52)
V_d_/F (L)	74.8 (16.2)	92.2 (35.7)	63.6 (15.6)	63.5 (16.5)
T_max_ (h)	0.94 (0.58–1.80)	0.96 (0.58–1.78)	0.83 (0.58–3.33)	0.84 (0.57–3.36)

Data are presented as the mean (standard error), except for Tmax (min–max). N = number of subjects; Cmax, maximum concentration; AUC_0–12h_, area under the concentration curve from zero to 12 h; CL/F, apparent clearance (dose/AUC_inf_); lambda z, terminal slope of the time-log-transformed concentration curve; half-life (h) = In(2)/lambda z; V_d_/F, apparent volume of distribution (CL/F/Lambda z); Tmax, time to reach the maximum concentration.

**Table 2:** Parameter estimates in the final pharmacokinetic model with bootstrap results.

Parameters	Estimates (RSE ^a^ %)	Bootstrap	
		Median	95% CI
Fixed effects			
K_a_ (1/h)	5.41 (45.47)	5.66	3.24–11.24
V_d_/F (L)	61.70 (5.82)	61.33	54.89–68.64
CL/F (L/h)	16.90 (8.40)	16.87	14.29–19.50
ALAG (h)	0.394 (6.24)	0.394	0.385–0.493
Interindividual variability (IIV)			
ω^2^_Ka_ (CV ^b^ %)	158.91 (43.65)	157.81	69.76–444.98
ω^2^_Vd_ (CV%)	22.10 (47.17)	20.98	12.0–29.68
ω^2^_CL_ (CV%)	32.60 (45.54)	31.74	20.96–41.65
ρ_Vd-CL_	0.853	0.853	0.796–0.869
Residual variability			
Proportional error	0.284 (6.16)	0.283	0.242–0.319

^a^ RSE (%), relative standard error (standard error/parameter estimate); ^b^ CV (%), coefficient of variance; K_a_, absorption rate constant; V_d_/F, apparent central volume of distribution; CL/F, apparent clearance; ALAG, absorption lag time.

**Table 3:** The percentage of the simulation number that reached toxic, comatose, and fatal concentration levels after zolpidem dosing.

	Doses (mg)	NR ^a^ (%)	<1 h (%)	1–2 h (%)	2–3 h (%)	>3 h (%)
Toxic concentration (500–1500 ng/mL)	10	100	0	0	0	0
	280	0	0	0	0	100
	560	0	0	0	0	100
	1120	0	0	0	0	100
Comatose concentration (1500–4000 ng/mL)	10	100	0	0	0	0
	280	0.2	0.4	0.8	15.0	83.6
	560	0	0	0	0.4	99.6
	1120	0	0	0	0	100
Fatal concentration (>4000 ng/mL)	10	100	0	0	0	0
	280	58.8	21.4	15.6	4.0	0.2
	560	2.6	1.6	11.2	31.4	53.2
	1120	0	0	0	1.6	98.4

^a^ NR, not reached. The percentages indicate the proportion of total simulations that were maintained within the toxic, comatose, and fatal concentration ranges after the administration of 10, 280, 560, and 1120 mg of zolpidem.

## Data Availability

The dataset used in this study is available from the corresponding author upon request.
